# Medical emergency team admittance to intensive care versus conventional admittance: characteristics and outcome

**DOI:** 10.1186/cc11114

**Published:** 2012-03-20

**Authors:** G Jäderling, M Bell, CR Martling, A Ekbom, M Bottai, D Konrad

**Affiliations:** 1 Karolinska Institutet, Stockholm, Sweden

## Introduction

The purpose of the medical emergency team (MET) is to find and treat deteriorating ward patients. Suboptimal care and delays on general wards before admission to intensive care have an effect on mortality [1] and patients admitted from general wards have a worse outcome than from the operating room (OR) or emergency department (ED) [2]. MET patients have a high rate of ICU admissions but whether their outcome differs from other patients admitted from the wards has not been studied before. We evaluated characteristics and outcome of ICU patients based on mode of admittance, via the MET versus the conventional way.

## Methods

An observational prospective study of patients admitted from general wards to the central ICU at Karolinska University hospital, Stockholm, Sweden in 2007 to 2009. Two groups were identified: admissions directly following a MET call or the conventional way, usually on request from the ward physician. Patients were analyzed for age, gender, co-morbidities, length of stay, severity scoring system (APACHE II) and mortality.

## Results

Of 2,571 ICU admissions, 694 admissions in 643 patients came from the wards. In total, 355 were admitted by the MET and 339 were conventional admissions. Median age was 65 years in the MET group versus 58 years in the conventional group, hospital LOS prior to ICU admission was median 3 days versus 1 day and APACHE II score was a mean of 26 versus 21. They did not differ as to proportion of invasive ventilator treatment or dialysis but MET patients more often received noninvasive ventilation, 57.2% versus 29.2% (*P *< 0.01). ICU mortality was 14.5% versus 8.9% (*P *= 0.04) and 30-day mortality 27.0% versus 19.1% (*P *= 0.02). MET patients also had a higher proportion of co-morbidities, with a prevalence of heart failure in 17.3% versus 11.7% (*P *= 0.0.4) and malignancy in 45.3% versus 35.1% (*P *< 0.01) as well as a higher proportion of limitation of medical treatment (LOMT), 23.0% versus 15.7% (*P *= 0.02). When LOMT patients were excluded, mortality rates were no longer significantly different, ICU mortality then being 5.7% versus 3.3% (*P *= 0.2).

## Conclusion

Two distinct groups of patients with intensive care needs are found in general wards. Those admitted by the MET are older, have more severe co-morbidities and have been in hospital longer. We find the MET to be an important tool to identify patients with multiple problems and at high risk of an adverse outcome.
